# Injury analysis of 257 hospitalized casualties of the Jishishan earthquake in Linxia Prefecture

**DOI:** 10.3389/fpubh.2025.1565550

**Published:** 2025-06-13

**Authors:** Xuequan Wei, Mingyan Ma, Zhanlin Zhang, Xiaozhong Li, Xinting Lu, Wenguang Pan, Yongdong An

**Affiliations:** People’s Hospital of Linxia Hui Autonomous Prefecture, Linxia, Gansu, China

**Keywords:** earthquake, Jishishan earthquake, earthquake casualties, injury characteristics, injury analysis

## Abstract

**Objective:**

To analyze the characteristics of injuries sustained by casualties of the Jishishan earthquake in Jishishan County, Linxia Hui Autonomous Prefecture, Gansu Province, China.

**Methods:**

A descriptive research approach was employed. Data were retrospectively collected and analyzed for earthquake-related injuries among patients admitted to the People’s Hospital of Linxia Hui Autonomous Prefecture, the People’s Hospital of Jishishan County, and the Traditional Chinese Medicine Hospital of Linxia Hui Autonomous Prefecture.

**Results:**

A total of 257 patients were hospitalized: 142 at the People’s Hospital of Linxia Hui Autonomous Prefecture, 91 at the People’s Hospital of Jishishan County, and 24 at the Traditional Chinese Medicine Hospital of Linxia Hui Autonomous Prefecture. Most injuries occurred during the main shock (96.5%). In total, 802 injuries were diagnosed, with fractures accounting for 46.6%, followed by visceral injuries (12.0%). The thorax was the most frequently affected site (25.8%), followed by the head (19.5%). Dislocations primarily involved the shoulder-upper arm (50.0%), whereas soft tissue contusions were most common in the lower back (20.3%). Skin lacerations were primarily located on the head (44.7%). Visceral injuries mainly affected the thorax (77.1%), with crush injuries concentrated in the hip-thigh region (38.5%). Hematomas and hemorrhages were predominantly seen in the head (87.0 and 76.0%, respectively). Single fractures were most frequent in the thorax (21.2%) and pelvis (15.2%). Pelvic fractures were the most common single-region multiple fractures (10.5%), whereas thorax+lower back injuries (13.7%) were the most frequent multiple-region fractures. Lung contusions constituted 67.0% of visceral injuries.

**Conclusion:**

In the Jishishan earthquake, pelvic and thorax fractures were observed as the main injuries, reflecting the interaction between the vertical collapse mechanism and the vulnerability of the buildings in the earthquake area. This study suggests the need to optimize the allocation of emergency resources for combined thorax-lower back-pelvic injuries in the plateau environment and to enhance the retrofitting of earthquake-resistant buildings in rural areas to reduce the incidence of such injuries. The core findings of this study support empirical evidence for the regional specificity of the earthquake injury spectrum, providing key evidence for regionalized prevention and treatment of earthquake trauma.

## 1 Introduction

Major natural disasters pose a significant threat to human life and health. Earthquakes, exemplify such natural disasters due to their abrupt onset, strong destructiveness, extensive social impact, potential for severe secondary disasters, and challenges associated with preparedness and mitigation, and once an earthquake occurs, it can inflict substantial damage on society (1, 2). The consequences of these earthquakes can be devastating, with statistics indicating that from 1900 to the end of the 20th century, over 500,000 people lost their lives due to seismic events in China, and this figure represents about half of the global fatalities attributed to earthquakes during that time period (3). It is evident that earthquake disasters are a critical challenge faced by Chinese society.

In the aftermath of an earthquake, scholars engage in timely analyses and discussions regarding the disaster’s impact on human society. They systematically evaluate and summarize the injuries to inform subsequent treatment practices. Analyses have been conducted on injury data from notable earthquakes, including the 7.6-magnitude quake in northern Pakistan in 2005, the 7.9-magnitude earthquake off the coast of Peru in 2007, the 7.2-magnitude earthquake in Haiti in 2010, and the 7.8-magnitude earthquake in Nepal in 2015 (4–7).

On December 18, 2023, at 23:59 Beijing time, a magnitude 6.2 earthquake struck Jishishan County at a depth of 10 km (8). Jieshishan County is on the transition zone between the Loess Plateau and the Tibetan Plateau, with an altitude of 1,787–4,308 meters (9). Located in the southeastern Ganshan region on the northeastern edge of the Tibetan Plateau, Jishishan County lies within a complex network of faults, influenced by the northeastern extension of the Tibetan Plateau (8). The region is characterized by the north edge and south edge of the Laji Mountain fault system, which runs from west to east, and the faults shift direction, turning southeast before intersecting with the Methodist South Mountain Fault and the north edge of the West Qinling Fault (10). This earthquake was classified as a shallow-source event and was characterized as a “recoil-type earthquake,” which typically results in stronger ground shaking. Consequently, the damage inflicted by this earthquake was relatively severe for its magnitude (11). Additionally, the Jishishan earthquake induced liquefaction of the saturated water loess layer beneath the plateau surface, triggering an unusual mudflow disaster in Zhongchuan Township, Qinghai Province (12). This earthquake resulted in 113 fatalities and 536 injuries (13).

A prospective cohort study of earthquake survivors in Padang, Indonesia, found that the physical damage caused by the earthquake could result in long-term disability and a decreased quality of life, and timely intervention and appropriate treatment may prevent death and disability by accurately characterizing the injuries (14). Consequently, the characteristics of the injuries sustained deserve thorough investigation and analysis. Since the occurrence of the Jishishan earthquake, there has been a scarcity of relevant studies addressing the injuries sustained during this event. Therefore, the objective of this study was to investigate the injuries and their characteristics among 257 hospitalized casualties admitted to the People’s Hospital of Linxia Hui Autonomous Prefecture, the People’s Hospital of Jishishan County, and the Traditional Chinese Medicine Hospital of Linxia Hui Autonomous Prefecture during the Jishishan earthquake. The findings aim to provide valuable insights and references for emergency rescue planning and to optimize treatment processes for earthquake casualties in similarly affected regions in the future.

## 2 Materials and methods

### 2.1 Source of information

This study received approval from the Ethics Committee of the People’s Hospital of Linxia Hui Autonomous Prefecture (LZYY-LLSP-2024-01). A retrospective analysis was conducted on the clinical records of 257 hospitalized casualties admitted to the People’s Hospital of Linxia Hui Autonomous Prefecture, the People’s Hospital of Jishishan County, and the Traditional Chinese Medicine Hospital of Linxia Hui Autonomous Prefecture following the 6.2 magnitude earthquake in Jishishan on December 18, 2023, and an electronic database was established.

### 2.2 Research and statistical methods

This study is descriptive in nature, and retrospectively collect information on the basic characteristics, distribution of injury diagnoses by body region, distribution of fracture injuries by body region, and diagnoses of visceral injuries among the 257 hospitalized earthquake casualties. The data analysis is mainly statistical description, and counting data is expressed by composition ratio. The Chi-square test was used for subgroup analysis of multiple injury and single injury with different demographic characteristics, and *p* < 0.05 was considered statistically significant.

### 2.3 Study site

The earthquake occurred in Jishishan County, Linxia Hui Autonomous Prefecture, Gansu Province, China. The casualties from the earthquake were primarily admitted to the general hospitals nearest to the epicenter: the People’s Hospital of Linxia Hui Autonomous Prefecture, the People’s Hospital of Jishishan County, and the Traditional Chinese Medicine Hospital of Linxia Hui Autonomous Prefecture. These three hospitals played a crucial role in treating and transferring injured individuals following the Jishishan earthquake. This study includes a total of 257 hospitalized casualties, comprising 142 patients admitted to the People’s Hospital of Linxia Hui Autonomous Prefecture, 91 patients at the People’s Hospital of Jishishan County, and 24 patients at the Traditional Chinese Medicine Hospital of Linxia Hui Autonomous Prefecture.

The People’s Hospital of Linxia Hui Autonomous Prefecture serves as the primary data collection site for this study. It is distinguished as the only Grade 3A general hospital in Linxia Prefecture and is the nearest facility equipped to treat critically ill patients affected by the earthquake in Jishishan County. The People’s Hospital of Jishishan County is classified as a Grade 2A general hospital and is located closest to the earthquake’s epicenter. The Traditional Chinese Medicine Hospital of Linxia Hui Autonomous Prefecture operates as a Grade IIIB hospital, integrating both traditional Chinese and Western medicine approaches in the context of the disaster.

### 2.4 Study variables

The basic characteristics included sex, age, marital status, ethnicity, occupation, and injury time of the earthquake victims. Age was recorded as a continuous variable in the original database, and according to the WHO’s age classification standard, age was classified into three categories: <18 years, 18 to 65 years, and >65 years. Marital status was categorized as single, married, divorced, and widowed. Ethnicity was classified into Han, Hui, Dongxiang, Baoan, Salar, Tu, Tibetan, and Tujia. Occupation was divided into farmer, student, self-employed, freelance, professional, retiree, and other. Injury time was classified as occurring during the main shock or the aftershock.

The injury category variable was derived from the discharge diagnoses recorded on the first page of the patients’ medical records. Diagnoses were coded according to the International Classification of Diseases (ICD)-10, classifying injuries as either single or multiple based on the diagnosis. Injury categories included fracture, dislocation, soft tissue contusion, skin laceration, visceral injury, crush injury, burn, nerve injury, hematoma, hemorrhage, and other trauma. Body regions were classified as head, eye-maxillofacial, neck, thorax, lower back, abdomen, pelvis, shoulder-upper arm, elbow-forearm, wrist-hand, hip-thigh, knee-lower leg, and ankle-foot. Fractures and visceral injuries were more prevalent in this study, and the body region distribution of fractures and the types of visceral injuries were analyzed separately.

### 2.5 Inclusion and exclusion criteria

#### 2.5.1 Inclusion criteria

(1) Jishishan earthquake casualties; (2) hospitalization; and (3) Injury information is complete.

#### 2.5.2 Exclusion criteria

(1) Injury information is incomplete; (2) Duplicate records, which to avoid multiple records for the same casualty; and (3) Non-earthquake related injuries.

## 3 Result

### 3.1 Distribution of basic characteristics of earthquake casualties

In this study, data from a total of 259 hospitalized earthquake casualties were collected from the People’s Hospital of Linxia Hui Autonomous Prefecture, the People’s Hospital of Jishishan County, and the Traditional Chinese Medicine Hospital of Linxia Hui Autonomous Prefecture. Of these, 2 casualties with incomplete injury information were excluded, and finally 257 casualties included in the analysis. Among these, 102 cases (39.7%) experienced single injuries, whereas 155 cases (60.3%) suffered multiple injuries. The gender distribution indicated a higher prevalence of females (53.7%) compared to males (46.3%), all sustaining multiple injuries. The age group most affected was 18 ~ 65 years (65.0%), with multiple injuries being prominent across all age groups. Marital status revealed a majority of married individuals (77.0%); among the widowed, single injuries were slightly more common for multiple injuries (54.6% vs. 45.5%), whereas multiple injuries predominated in all other marital statuses. In terms of ethnicity, the Hui group represented the largest proportion (47.5%), with Tibetan and Tujia individuals primarily sustaining single injuries, whereas other ethnic groups experienced multiple injuries. Most of the injured were farmers (75.9%), with freelance and retired individuals having only single injuries; all other occupations sustained multiple injuries. The majority of injuries occurred during the main shock (96.5%). The subgroup analysis of multiple injury and single injury with different demographic characteristics showed that there were no statistical significance (*p* > 0.05) (Table 1).

**Table 1:** Distribution of basic characteristics of earthquake casualties [*n* (%)].

Variables		Total	Single injury	Multiple injuries
		*N* = 257	*n* = 102	*n* = 155
Sex	Male	119 (46.3%)	43 (42.2%)	76 (49.0%)
	Female	138 (53.7%)	59 (57.8%)	79 (51.0%)
Age	<18	41 (16.0%)	18 (17.6%)	23 (14.8%)
	18–65	167 (65.0%)	66 (64.8%)	101 (65.2%)
	>65	49 (19.0%)	18 (17.6%)	31 (20.0%)
Marital status	Single	43 (16.7%)	19 (18.6%)	24 (15.5%)
	Married	198 (77.0%)	75 (73.5%)	123 (79.4%)
	Divorced	5 (2.0%)	2 (2.0%)	3 (1.9%)
	Widowed	11 (4.3%)	6 (5.9%)	5 (3.2%)
Nationality	Han	81 (31.5%)	33 (32.4%)	48 (31.0%)
	Hui	122 (47.5%)	45 (44.1%)	77 (49.7%)
	Dongxiang	16 (6.2%)	8 (7.8%)	8 (5.2%)
	Baoan	17 (6.6%)	7 (6.8%)	10 (6.4%)
	Salar	14 (5.4%)	5 (4.9%)	9 (5.8%)
	Tu	5 (2.0%)	2 (2.0%)	3 (1.9%)
	Tibetan	1 (0.4%)	1 (1.0%)	0 (0%)
	Tujia	1 (0.4%)	1 (1.0%)	0 (0%)
Occupation	Farmer	195 (75.9%)	73 (71.6%)	122 (78.7%)
	Student	28 (10.9%)	13 (12.7%)	15 (9.7%)
	Self-employed	5 (2.0%)	1 (1.0%)	4 (2.6%)
	Freelance	4 (1.5%)	4 (3.9%)	0 (0%)
	Professional	3 (1.2%)	1 (1.0%)	2 (1.3%)
	Retired	2 (0.7%)	2 (2.0%)	0 (0%)
	Other	20 (7.8%)	8 (7.8%)	12 (7.7%)
Injury time	Main shock	248 (96.5%)	96 (94.1%)	152 (98.1%)
	Aftershock	9 (3.5%)	6 (5.9%)	3 (1.9%)

The above subgroup analysis showed *p* > 0.05.

### 3.2 Distribution of body regions for injury diagnosis in earthquake casualties

A total of 802 injuries were diagnosed in 257 patients. Fractures were the most common (46.6%), followed by visceral injury (12.0%), other trauma (10.9%), soft tissue contusion (9.9%), hematoma (8.6%), skin laceration (5.9%), hemorrhage (3.1%), crush injury (1.6%), dislocation (1.0%), and burn and nerve injury (0.2% each). Based on the distribution of body regions, injuries were most frequently observed in the thorax (25.8%), followed by the head (19.5%), pelvis (12.0%), lower back (9.0%), eye-maxillofacial region (7.4%), ankle-foot (5.3%), hip-thigh (4.4%), abdomen (4.2%), shoulder-upper arm (3.9%), elbow-forearm (3.4%), knee-lower leg (3.1%), wrist-hand (1.9%), and neck (0.1%) (Table 2).

**Table 2:** Distribution of body regions for injury diagnosis in earthquake casualties.

Variables	Head	Eye-maxillofacial	Neck	Thorax	Lower back	Abdomen	Pelvis	Shoulder-upper arm	Elbow- forearm	Wrist- hand	Hip-thigh	Knee-lower leg	Ankle-foot	Total [*n* (%)]
Fracture	19	23	1	97	49	0	79	19	18	3	20	14	32	374 (46.6%)
Dislocation	0	0	0	0	3	0	0	4	0	0	1	0	0	8 (1.0%)
Soft tissue contusion	6	9	0	1	16	11	4	5	3	7	7	6	4	79 (9.9%)
Skin laceration	21	13	0	0	0	0	1	1	4	4	0	0	3	47 (5.9%)
Visceral injury	0	0	0	74	0	15	5	0	0	0	2	0	0	96 (12.0%)
Crush injury	0	0	0	0	2	0	0	0	0	0	5	3	3	13 (1.6%)
Burn	0	0	0	0	0	1	0	0	1	0	0	0	0	2 (0.2%)
Nerve injury	0	0	0	0	0	0	0	1	1	0	0	0	0	2 (0.2%)
Hematoma	60	1	0	0	0	3	4	0	0	0	0	1	0	69 (8.6%)
Hemorrhage	19	2	0	0	0	3	1	0	0	0	0	0	0	25 (3.1%)
Other trauma	31	11	0	35	2	1	2	1	0	1	0	1	2	87 (10.9%)
Total [*n* (%)]	156 (19.5%)	59 (7.4%)	1 (0.1%)	207 (25.8%)	72 (9.0%)	34 (4.2%)	96 (12.0%)	31 (3.9%)	27 (3.4%)	15 (1.9%)	35 (4.4%)	25 (3.1%)	44 (5.3%)	802 (100.0%)

Fractures were noted in all body regions except the abdomen, with the majority occurring in the thorax (25.9%), followed by the pelvis (21.1%), and lower back (13.1%). Dislocations were predominantly observed in the shoulder-upper arm (50.0%), lower back (37.5%), and hip-thigh (12.5%). Soft tissue contusions affected all body regions except the neck, with the majority occurring in the lower back (20.3%), abdomen (13.9%), and eye-maxillofacial region (11.4%). Skin lacerations were most common in the head (44.7%) and eye-maxillofacial region (27.7%), followed by the elbow-forearm and wrist-hand (8.5% each), ankle-foot (6.4%), pelvis, and shoulder-upper arm (2.1% each). Visceral injuries were predominantly found in the thorax (77.1%), followed by the abdomen (15.6%), pelvis (5.2%), and hip-thigh (2.1%). Crush injuries were most prevalent in the hip-thigh (38.5%), followed by the knee-lower leg and ankle-foot (23.1% each), and lower back (15.4%). Burns were most frequently reported in the abdomen and elbow-forearm (50.0% each). Nerve injuries were primarily seen in the shoulder-upper arm and elbow-forearm (50.0% each), whereas hematomas were chiefly located in the head (87.0%), followed by the pelvis (5.8%), abdomen (4.3%), and knee-lower leg and eye-maxillofacial region (1.5% each). Hemorrhages predominantly affected the head (76.0%), followed by the abdomen (12.0%), eye-maxillofacial region (8.0%), and pelvis (4.0%). Other trauma cases primarily occurred in the thorax (40.2%) and head (35.6%) (Table 2).

### 3.3 Distribution of body regions of 99 casualties involving single fracture

In this study, the injury diagnoses of 194 patients included fractures, of which 99 were classified as single fractures (51.0%) and 95 as multiple fractures (49.0%). The most common site for a single fracture was the thorax (21.2%), followed by the pelvis (15.2%), lower back (12.1%), ankle-foot (11.1%), head and knee-lower leg (9.1% each), hip-thigh (8.1%), elbow-forearm (7.1%), shoulder-upper arm (4.0%), eye-maxillofacial region (2.0%), and wrist-hand (1.0%) (Table 3).

**Table 3:** Distribution of body regions of 99 casualties involving single fracture.

Variables	*n* (%)
Head	9 (9.1%)
Eye-maxillofacial	2 (2.0%)
Thorax	21 (21.2%)
Lower back	12 (12.1%)
Pelvis	15 (15.2%)
Shoulder-upper arm	4 (4.0%)
Elbow-forearm	7 (7.1%)
Wrist-hand	1 (1.0%)
Hip-thigh	8 (8.1%)
Knee-lower leg	9 (9.1%)
Ankle-foot	11 (11.1%)

### 3.4 Distribution of body regions of 95 casualties involving multiple fractures

There were 21 patients (22.1%) who presented with multiple fractures in a single body region, whereas 74 patients (77.9%) exhibited fractures across multiple body regions. The pelvis emerged as the most frequently affected single body region, accounting for 10.5% of the cases. This was followed by the thorax (4.2%), the elbow-forearm, and the ankle-foot (3.2% each), with the knee-lower leg representing 1.1%.

The majority of fractures involving multiple body regions were observed in combinations such as the thorax+lower back, which constituted 13.7% of cases. Other notable combinations included the thorax+pelvis (11.6%), shoulder-upper arm+thorax (4.2%), shoulder-upper arm+thorax+pelvis (4.2%), head+eye-maxillofacial injuries (3.2%), thorax+lower back+ pelvis (3.2%), as well as eye-maxillofacial+lower back injuries (2.1%). Additionally, combinations such as thorax+ankle-foot (2.1%), knee-lower leg+ankle-foot (2.1%), along with other injury regions, each represented 1.1% (refer to Table 4).

**Table 4:** Distribution of body regions of 95 casualties involving multiple fractures.

Variables	*n* (%)
Single body region	
Thorax	4 (4.2%)
Pelvis	10 (10.5%)
Elbow-forearm	3 (3.2%)
Knee-lower leg	1 (1.1%)
Ankle-foot	3 (3.2%)
Multiple body regions	
Head+Thorax	1 (1.1%)
Head+Eye-maxillofacial	3 (3.2%)
Eye-maxillofacial+Lower back	2 (2.1%)
Eye-maxillofacial+Hip-thigh	1 (1.1%)
Neck+Thorax	1 (1.1%)
Thorax+Pelvis	11 (11.6%)
Thorax+Lower back	13 (13.7%)
Thorax+Hip-thigh	1 (1.1%)
Thorax+Ankle-foot	2 (2.1%)
Lower back+Pelvis	1 (1.1%)
Lower back+Hip-thigh	1 (1.1%)
Lower back+Wrist-hand	1 (1.1%)
Pelvis+Hip-thigh	1 (1.1%)
Pelvis+Knee-lower leg	1 (1.1%)
Shoulder-upper arm+Thorax	4 (4.2%)
Shoulder-upper arm+Pelvis	1 (1.1%)
Elbow-forearm+Thorax	1 (1.1%)
Elbow-forearm+Ankle-foot	1 (1.1%)
Hip-thigh+Ankle-foot	1 (1.1%)
Knee-lower leg+Ankle-foot	2 (2.1%)
Head+Thorax+Knee-lower leg	1 (1.1%)
Eye-maxillofacial+Shoulder-upper arm+Thorax	1 (1.1%)
Eye-maxillofacial+Thorax+Pelvis	1 (1.1%)
Eye-maxillofacial+Thorax+Hip-thigh	1 (1.1%)
Eye-maxillofacial+Thorax+Lower back	1 (1.1%)
Thorax+Pelvis+Hip-thigh	1 (1.1%)
Thorax+Lower back+Pelvis	3 (3.2%)
Thorax+Lower back+Ankle-foot	1 (1.1%)
Thorax+Lower back+Hip-thigh	1 (1.1%)
Lower back+Pelvis+Hip-thigh	1 (1.1%)
Lower back+Shoulder-upper arm+Wrist-hand	1 (1.1%)
Shoulder-upper arm+Thorax+Pelvis	4 (4.2%)
Pelvis+Hip-thigh+Ankle-foot	1 (1.1%)
Head+Shoulder-upper arm+Thorax+Hip-thigh	1 (1.1%)
Eye-maxillofacial+Shoulder-upper arm+Thorax+Pelvis	1 (1.1%)
Eye-maxillofacial+Shoulder-upper arm+Thorax+Lower back	1 (1.1%)
Shoulder-upper arm+Thorax+Lower back+Hip-thigh	1 (1.1%)
Eye-maxillofacial+Thorax+Lower back+Pelvis+Hip-thigh	1 (1.1%)
Elbow-forearm+Thorax+Lower back+Pelvis+Ankle-foot	1 (1.1%)

### 3.5 Injury diagnosis in 85 casualties involving visceral injury

In this study of 85 patients with visceral injuries, 72 patients (84.7%) sustained single injuries, whereas 13 patients (15.3%) experienced multiple injuries. Lung contusion was identified as the most prevalent single visceral injury, accounting for 67.0% of cases. Follow by the chest injuries constituted 7.0%, traumatic pleural effusion made up 4.7%, and splenic rupture accounted for 2.3%. Mesentery contusion, bladder rupture, and liver contusion were each observed in 1.2% of cases.

Among those with multiple injuries, the most common combinations included 3.5% for lung contusion+ splenic rupture, and 2.3% for lung contusion+traumatic pleural effusion. Additionally, 1.2% of cases involved combinations of the following injuries: chest injury+traumatic pleural effusion, traumatic pneumothorax+traumatic pleural effusion, rupture of left testis+Laceration of the scrotum, lung contusion+bladder injury, lung contusion+hepatic rupture, lung contusion+bladder rupture+adrenal hematoma, lung contusion+liver contusion+kidney contusion, and splenic rupture+liver contusion+bladder contusion+uterine contusion (Table 5).

**Table 5:** Injury diagnosis in 85 casualties involving visceral injuries.

Variables	*n* (%)
Single injury diagnoses	
Mesentery contusion	1 (1.2%)
Bladder rupture	1 (1.2%)
Splenic rupture	2 (2.3%)
Liver contusion	1 (1.2%)
Chest injury	6 (7.0%)
Traumatic pleural effusion	4 (4.7%)
Lung contusion	57 (67.0%)
Multiple injury diagnoses	
Chest injury+Traumatic pleural effusion	1 (1.2%)
Traumatic pneumothorax+Traumatic pleural effusion	1 (1.2%)
Rupture of left testis+Laceration of the scrotum	1 (1.2%)
Lung contusion+Bladder injury	1 (1.2%)
Lung contusion+Hepatic rupture	1 (1.2%)
Lung contusion+Splenic rupture	3 (3.5%)
Lung contusion+Traumatic pleural effusion	2 (2.3%)
Lung contusion+Bladder rupture+Adrenal hematoma	1 (1.2%)
Lung contusion+Liver contusion+Kidney contusion	1 (1.2%)
Splenic rupture+Liver contusion+Bladder contusion+Uterine contusion	1 (1.2%)

## 4 Discussion

The Jishishan earthquake struck during the late night in the dead of winter, and research indicates that the number of casualties resulting from earthquakes occurring at night is significantly higher compared to those occurring during the day, even when accounting for similar magnitudes (15). Furthermore, the high population density and the substantial proportion of buildings with inadequate seismic capacity in the affected area were key factors contributing to the significant number of casualties (12). In this study, it was found that multiple injuries comprised 60.3% of earthquake-related casualties. This data suggests a predominance of multiple injuries among the victims of the Jishishan earthquake, which aligns with findings from Zhao (16) regarding the characteristics of injuries sustained during the Wenchuan earthquake. These results highlight the necessity for multidisciplinary and coordinated treatment in the aftermath of such disasters. Likewise, Selahattin Gürü’s findings underscore the importance of strong multidisciplinary treatment preparedness in areas prone to natural disasters (17).

According to the China News Network, the Jishishan earthquake was followed by a total of 423 aftershocks, the largest registering a magnitude of 4.1 (18). The findings of this study revealed that the majority of injuries occurred during the main shock, with multiple injuries predominating at this time. In contrast, single injuries were more commonly reported during the aftershocks. This distribution may be attributed to the greater magnitude and destructive impact of the main shock compared to the aftershocks, as well as the fact that many individuals had already been relocated following the initial event.

In this study, a total of 802 injuries were diagnosed among 257 patients, with fractures being the most prevalent injury at 46.6%, followed by visceral injuries at 12.0%. The majority of injuries occurred in the thorax, with the head being the second most affected area. Zhao (16) reported that 3,177 injuries were diagnosed in 1,871 casualties from the Wenchuan earthquake, where fractures also constituted the majority at 46.5%, primarily affecting the head (22.9%). Similarly, Peng (19) examined 2,622 casualties from the Yushu earthquake and found that fractures were the most common injury, accounting for 55.08%, with injury locations predominantly in the lower limbs and pelvis (36.0%), followed by the chest (13.3%). In another study, Peng (7) identified 521 injuries among 266 earthquake casualties in Lushan, with fractures again being the most common at 41.5%, followed by soft tissue injuries at 27.5%, and injuries were primarily concentrated in the lower limbs and pelvis (34.2 and 17.9%, respectively). These findings indicate that fractures are the most common type of injury observed in several major earthquakes in our country over recent years. This suggests a need for enhanced allocation of orthopedic personnel and related medical resources during earthquake emergency response to ensure that the injured receive timely and effective treatment. However, the common injury sites vary, potentially due to the timing of the earthquakes and various factors, such as individuals’ exposure and body posture at the time of the events (20). Notably, the Wenchuan, Yushu, and Lushan earthquakes occurred during the daytime, whereas the Jishishan earthquake struck at 23:59, when most individuals were sleeping. Moreover, the power outage caused by the earthquake may have hindered timely evasion responses.

Some scholars have proposed that if a patient is standing or sitting during an earthquake, the spinal region is most likely to sustain fractures, conversely, if the patient is in a supine or lateral position, the pelvis and thorax are more commonly affected (21). In this study, single fractures slightly outnumbered multiple fractures (51.0% vs. 49.0%), with single fractures primarily affecting the thorax. The pelvis was the most common site for single body regions with multiple fractures, whereas multiple body regions primarily involved the thorax+lower back. Additionally, the study noted that dislocations were rare, comprising only 1% of cases, and predominantly affected the shoulder-upper arm and lower back. Previous research has indicated that in earthquakes in northern Pakistan (4), Kahramanmaraş (22), Wenchuan (16, 23) and Yushu (19), the lower limbs were the primary fracture sites, whereas in Lushan (7), both the lower limbs and pelvis were commonly affected. The main reason for these differences may be that the Jishishan earthquake occurred late at night when people were typically asleep, often lying on their backs or sides.

In this study, soft tissue contusions accounted for 9.9% of injuries and involved all body areas except the neck, with the lower back being the most affected region. In contrast, patients from the Wenchuan earthquake primarily experienced soft tissue injuries in the chest (16), indicating that different exposure conditions may account for the variation in primary injury sites. Burn injuries represented only 0.3% of cases in this study, compared to 1.7% in the Lushan earthquake (7). Although stoves were commonly used for heating and cooking in both regions, the relatively low incidence of burn injuries in the Jishishan event can largely be attributed to the timing of the earthquake, which occurred late at night whereas individuals were asleep and situated further away from the stoves.

Crush injuries, resulting from direct physical trauma and compression, are often seen in the lower limbs, upper limbs, and trunk during earthquakes (24). In this study, crush injuries accounted for 1.6% of cases, with the lower limbs and lower back being the most affected. Comparatively, crush injuries among Wenchuan earthquake patients represented 3.5%, typically occurring in the lower legs and trunk (16). Despite similarities in the main injury sites between the two earthquakes, the lower incidence of crush injuries in the Jishishan earthquake may be attributed to its relatively low magnitude. The prevalence of visceral injury in this study was the second highest observed, with the thorax region being the most affected site. Lung contusion was the most frequent injury, occurring in 67.0% of cases. One patient presented with four internal injuries, which included splenic rupture, liver contusion, bladder contusion, and uterine contusion, highlighting the severity of internal injuries caused by the earthquake. However, prior studies related to the earthquakes in Wenchuan (16, 21), Yushu (19), Lushan (7), and others did not provide clear distributions of visceral injuries. The timing of the earthquake, occurring in the middle of the night whereas most individuals were asleep and in a supine or lateral position, contributed to the likelihood of sustaining internal injuries. Additionally, injuries from collapsing walls and roofs were significant contributors to visceral injuries. The earthquake predominantly affected ethnic regions, including Hui, Baoan, Salar, and Dongxiang communities, the architectural styles in these areas displayed distinct ethnic characteristics, primarily consisting of brick and wood, civil engineering, wood structure, and brick-concrete. Insufficient structural measures, poor construction quality, and issues with building materials were key factors contributing to the collapse of these buildings (12).

In response to the earthquake, and in alignment with the directives from the Party Central Committee, the State Council, and various governmental bodies, emergency response plans were swiftly activated. Medical personnel were mobilized to the disaster area to rescue and transport the injured. Fire and rescue teams also initiated a first-level response, focusing on search and rescue operations alongside fire safety measures. The state deployed rescue teams to oversee the treatment of the injured. Throughout the medical treatment process, a commitment to scientific, systematic, and standardized care was maintained, emphasizing the “concentration of patients, experts, resources, and centralized treatment.” All injured individuals were categorized for treatment based on the severity and urgency of their conditions. Following the earthquake, all relevant departments proactively advanced recovery and reconstruction efforts.

Two cases with incomplete data were excluded from this study, which may have had the following effects: if the excluded cases belonged to special injury types (e.g., rare compound injuries) or extreme degree of severity, it may have slightly affected the accuracy of the distribution of injury types; and considering the fact that the exclusion proportion was less than 1% and the admission criteria were consistent across the three hospitals, the impact of this missing data on the overall study findings could be contained within acceptable limits. A sensitivity analysis was performed to suggest that we recalculate the main indicators by assuming that the excluded cases belonged to the two cases of the lightest and the most severe injuries, respectively, and found that the differences in the results were minimally different, indicating that the study conclusions were robust. Future studies could further reduce the rate of missing information by establishing a multicenter trauma registry with standardized data collection forms.

The following experiences and insights have been summarized in the aftermath of the recent earthquake: Enhance the emergency response plan for public health emergencies, specifically focusing on earthquakes. Strengthen emergency drills to bolster response capabilities. Relevant government departments should coordinate the demolition of structures identified as potential safety hazards and improve the earthquake resilience of housing stock. Implement health education initiatives to raise public awareness about earthquakes, enabling individuals to respond appropriately and minimize harm during aftershocks. Ensure that households maintain readily accessible emergency power supplies to mitigate the impact of potential power outages following an earthquake. Expedite the treatment of injuries sustained during an earthquake, which includes on-site first aid, swift transfer of victims to the nearest medical facilities, and rapid pre-screening and classification at healthcare institutions. The medical treatment process should adhere to the principle of “focusing on patients, focusing on experts, focusing on resources, and focusing on treatment,” and should be categorized and centralized, facilitating improved efficiency and reducing mortality and morbidity among the injured. Recognizing that the loss of loved ones during earthquakes can lead to psychological trauma, it is crucial to enhance psychological support services for those affected.

Recommendations for multidisciplinary care needed for earthquake-related trauma cases. The multidisciplinary team is composed of the following departments. Department of Trauma Surgery: leading injury control surgery and life support; Department of Orthopedics: managing fractures; Department of Neurosurgery/Cerebrovascular Disease: spinal cord injuries and intracranial pressure management; Department of Critical Care Medicine: treatment and management of critically ill patients; Department of Pediatrics: management of injuries to children and infants; Department of Obstetrics and Gynecology: maternal care management; Department of Infection: development of antibiotic laddering protocols and prevention and control of multidrug-resistant organisms; Department of Rehabilitation Medicine: neuromuscular function restoration Department of Mental Health: screening and intervention for post-traumatic stress disorder (PTSD) and various psychological disorders; Department of Nutrition: development of high-protein metabolic support programs; Medical and Nursing Department: coordination of medical resources. Adopting the SBAR (Situation-Background-Assessment-Recommendation) model for Daily multidisciplinary rounds. Implementing an integrated electronic medical record (EMR) system with real-time synchronization of imaging, laboratory data and rehabilitation progress through integrated electronic medical records enables digital information sharing. Staged multidisciplinary treatment program, acute phase (within 72 h after the disaster), with damage control and life support and infection prevention and control. In the subacute phase (72 h-1 week), definitive surgical treatment and neurological function protection are the main focus. The rehabilitation period (after 1 week) focuses on neuromuscular rehabilitation and chronic pain management. Attention is also given to psychological intervention and treatment.

This study bears inherent methodological constraints associated with its retrospective design. The sample derivation from three tertiary hospitals introduces susceptibility to selection bias, particularly regarding the over representation of patients requiring critical care transfers. Furthermore, the exclusion of pre-hospital fatalities creates a truncated injury spectrum representation, potentially compromising epidemiological completeness. The absence of longitudinal follow-up mechanisms precludes robust analysis of chronic disability trajectories and delayed sequelae. Future multicenter prospective studies are needed to integrate physiological, environmental, and cultural multidimensional data to improve the generalizability of the findings. The establishment of regional trauma registries with embedded long-term surveillance systems could enable predictive modeling frameworks for post-disaster complication risks.

## 5 Conclusion

In the Jishishan earthquake, pelvic and thorax fractures were observed as the main injuries, reflecting the interaction between the vertical collapse mechanism and the vulnerability of the buildings in the earthquake area. This study suggests the need to optimize the allocation of emergency resources for combined thorax-lower back-pelvic injuries in the plateau environment and to enhance the retrofitting of earthquake-resistant buildings in rural areas to reduce the incidence of such injuries. Despite the limitations of retrospective data and sample selection bias, this study provides key evidence for regionalized prevention and treatment of earthquake trauma. Future research needs to join forces with emergency management departments to obtain data on prehospital deaths, conduct community sampling surveys, and collect information on minor injuries that did not seek medical attention to improve the completeness of the injury profile. Multi-center prospective cohort and molecular mechanism studies are needed to further reveal the regulatory pathways of the plateau environment on trauma prognosis. A trauma registry system covering the entire chain should be constructed, integrating prehospital, in-hospital and rehabilitation data, and machine learning models should be used to dynamically predict prognosis.

## Data Availability

The raw data supporting the conclusions of this article will be made available by the authors, without undue reservation.
